# Anti-Gravity Treadmill Training for Freezing of Gait in Parkinson’s Disease

**DOI:** 10.3390/brainsci10100739

**Published:** 2020-10-15

**Authors:** José Fidel Baizabal-Carvallo, Marlene Alonso-Juarez, Robert Fekete

**Affiliations:** 1 Department of Sciences and Engineering, University of Guanajuato, Guanajuato 37150, Mexico; 2 National Polytechnic Institute, Mexico City 07320, Mexico; marlenalonso@hotmail.com; 3 Department of Neurology, New York Medical College, Valhalla, NY 10595, USA; robertfekete@hotmail.com

**Keywords:** Parkinson’s disease, neurodegenerative disorders, freezing of gait, neurorehabilitation, rehabilitation, therapy

## Abstract

Background: Parkinson’s disease (PD) is a neurodegenerative disorder characterized by motor impairment. Freezing of gait, impaired mobility and falls are common problems in these patients. We aimed to evaluate the effect of a novel therapy for these patients. Methods: We studied patients with moderate to severe freezing of gait who underwent antigravity treadmill training twice a week for 4 consecutive weeks with 50% reduction of body weight. Results: We enrolled 26 consecutive patients with PD, 19 completed the study. There were 10 males; mean age at evaluation was 72.7 ± 10.1 years. Compared to baseline, patients showed improvement in the Freezing of Gait Questionnaire (*p* = 0.001); and a mean reduction of 7 s in the Timed Up & Go (TUG) test (*p* = 0.004). Moderate or significant improvement in gait was reported by 84% of patients. Conclusions: Antigravity treadmill training improved freezing of gait and mobility in patients with PD.

## 1. Introduction

Parkinson’s disease (PD) is a neurodegenerative disorder characterized by the presence of bradykinesia, muscle rigidity, tremor and abnormal postural reflex [1]. A substantial proportion of patients with PD have balance problems and freezing of gait (FoG) has been reported in 47% of cases [1]. FoG is defined as “brief, episodic absence or marked reduction of forward progression of the feet despite the intention to walk” and typically occurs on initiating gait or on turning while walking [2]. Several therapeutic approaches have been employed in clinical studies and medical practice in order to improve FoG, including: pharmacological therapy; brain, spinal cord and vagus nerve stimulation; and physiotherapeutic approaches using wearable cueing devices, treadmill training, walk-bicycle, or obstacle aquatic training with variable clinical benefit [3]. Despite the wide number of proposed therapies, FoG is still difficult to treat because many patients do not have a complete response to dopaminergic therapy [2] and neurostimulation techniques, although promising, may require invasive and expensive technology not always available [3]. In this scenario, physiotherapeutic approaches may be easier to apply with a lower risk of side effects and potential cardiovascular benefits.

In this study, we aimed to assess whether training with an antigravity treadmill can improve FoG and mobility in patients with PD. The antigravity treadmill technique was developed by the National Aeronautics and Space Administration (NASA) as a method for astronauts to maintain conditioning in space [4]. The available technology allows patients to walk with a programmed reduction of body weight, decreasing joint pressure in the lower limbs, making it suitable for patients with osteoarthritis and muscular deconditioning while preserving gait mechanics [5]. We hypothesized that patients with PD undergoing training under much lower body weight would decrease FoG and improve gait as previously reported in patients undergoing rehabilitation in low gravity environments such as aquatic therapy [6,7].

## 2. Materials and Methods

We enrolled patients diagnosed with PD according to the Queen Square Brain Bank Criteria [8] and Freezing of Gait Questionnaire (FoGQ) with a score equal or higher than 8 points, after optimizing dopaminergic therapy between 1 and 2 months prior to starting the therapy. Patients were enrolled in a single tertiary care clinic for movement disorders from August 2018 to January 2020. We excluded patients confined to a wheelchair, those with comorbid neurological disorders affecting gait other than PD and those with cardiovascular disorders precluding physical effort. Clinical evaluations were performed by a movement disorders specialist with part III (motor score) of the Movement Disorders Society Unified Parkinson’s Disease Rating Scale (MDS-UPDRS-III); the disease stage was assessed according to the modified Hoehn–Yahr scale [9]. The cognitive status was assessed by means of the Montreal Cognitive Assessment (MoCA). The FoGQ was used as inclusion criteria and to assess the severity and functional impact of FoG [10]. This clinical scale is composed by 6 items, each one of them rating the impairment between 0 (normal or lack of abnormality) to 4 (severely abnormal). Furthermore, item 2.13 of the MDS-UPDRS part II was also included to estimate the severity of FoG. The clinical response of FoG to levodopa was classified as: (1) unknown or unclear: including patients that have never taken the medication; (2) partially sensible; (3) resistant: no changes with any dose of levodopa; and (4) induced: worsening with levodopa [11].

The Timed Up & Go (TUG) test was carried out in order to assess the patient’s mobility, dynamic balance and fall risk [12]. Patients performed three TUG attempts, they were allowed to use a walking aid (walker, or cane) and be accompanied (not assisted) by their caregiver during the test, if necessary, to protect patients from injury in case of falling during the test. An averaged TUG test was obtained from the second and third attempts. The TUG test is reliable and does not seem to suffer from ceiling or floor effects [12]. Although patients and examiners were not blinded, they were unaware of the scores of baseline evaluations. The Clinical Global Impressions Scale (CGIS) was used to evaluate “severity” of the gait disorder at baseline and “improvement” after concluding all training sessions. Additionally, we asked patients whether they perceived improvement in equilibrium while walking, increased gait speed, longer steps and less FoG episodes after concluding the therapy. Finally, the number of falls in the month preceding and during the therapy was recorded.

The aforementioned clinical evaluations were carried out at baseline 48 to 96 h before starting the training sessions. Follow-up evaluations were performed between 24 and 72 h after the last training session. Medications for PD were not modified during the study period and all assessments were performed with patients in the medication “On” state. The study protocol received final approval by our local committee of ethics on March 2020 without modifications (Santé Medical Tower, no 202003-002) and patients or a close family member provided written informed consent to participate in the study.

**Figure 1:**
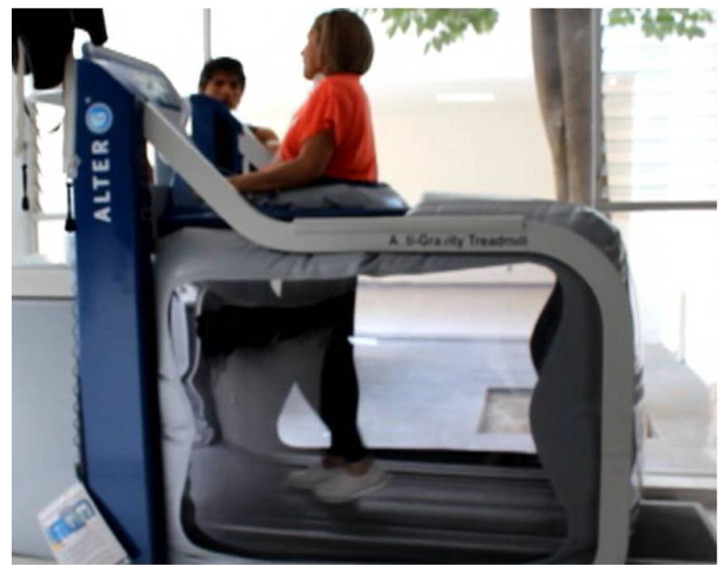
This picture shows the function of the antigravity treadmill (AlterG) which is based on positive air pressure in the lower extremities.

Procedure: Patients were instructed to take their anti-parkinsonian drugs at least one to two hours before each training session in order to perform it in the medication “On” state. Therapy was carried out with anti-gravity treadmill model M320 (AlterG, Inc., Fremont, CA, USA). The device can provide up-to 80% body-weight reduction by applying positive air pressure around the lower extremities during treadmill training (Figure 1). The AlterG M320 was cleared by the Food and Drug Administration (FDA) for use in medical and physical therapy clinics [4]. This technology has shown safety in systemic and intracranial hemodynamic parameters [13]. Patients underwent two training sessions per week over 4 consecutive weeks. Each session lasted up to 60 min according to patient’s tolerance. All patients underwent automatic weight estimation by the machine, followed by a programmed body-weight reduction of 50% in all sessions. The treadmill velocity was adapted depending on the individual tolerance on each session, but all patients were able to tolerate progressively faster velocities and training time. Baseline and follow-up evaluations were performed in the medication “On” state, 1 to 2 h following the last dose of dopaminergic medication, as patients underwent therapy in the “On” state and some of them were too impaired to carry out the evaluation in the medication “Off” state.

Data were summarized in means ± standard deviations, range, and percentages. The paired *t*-test was used to compare means before and after concluding the therapy. The McNemar’s test and Fisher’s exact test were used to compare nominal or ordinal data before and after the therapy. All statistical evaluations were performed using SPSS version 22 (Armonk, NY, USA), a *p* value < 0.05 was considered significant. Patients completing less than 70% of programmed sessions were eliminated from the analysis.

## 3. Results

### 3.1. Patients Included in the Cohort

Among 60 consecutive patients with PD, 27 patients fulfilled diagnostic criteria for inclusion and accepted to participate in the study. Among these patients, eight were not included in the final analysis: five patients did not finish the required number of training sessions (n = 2 lived far from the center; n = 1 had orthopedic problems, n = 1 argued social commitments and n = 1 was for unclear reasons) and three never started the therapy (n = 1 had hip problems; n = 1 lived far from the center; n = 1 died due to complications of Crohn’s disease). Patients not included in the analysis had a longer evolution time of PD compared to included patients 6.8 vs. 16.2 years (*p* = 0.011); no statistically significant differences were observed in other demographics, motor status, FoG and cognitive variables.

### 3.2. Clinical Features of Included Patients and Effect of the Therapy

A total of 19 patients were included in the analysis; there were 10 males with a mean age at evaluation of 72.7 ± 10.1 years; FoG was partially sensible to levodopa in 63.2% of patients; other clinical features are summarized in Table 1. Most patients (21 out of 26) required adjustment of dopaminergic medication with increased dose.

When comparing baseline to follow-up scores, an improvement in the total FOGQ score was observed (*p* = 0.001); although individual improvements were observed in all FOGQ sub-scores, difficulties with daily activities, independence and duration of freezing when turning did not reach statistical significance (Table 2). FoG scores also improved when assessed by items 2.13, 3.10 and 3.11 of the MDS-UPDRs-II and III, respectively. A decrease in recorded time was registered in the TUG test with an improvement of 7 sec when comparing mean times of the second and third attempts. Three patients were accompanied by caregivers in order to complete the TUG test. Falls were less common during therapy compared to the preceding months without reaching statistical significance.

A total of 16 (84%) patients self-rated their gait as “moderately better” or “much better” at the end of therapy and the majority of patients reported benefit in gait balance, as they stated that their gait was more secure, but also noted larger steps, increased gait speed, and perceived improvement in FoG (Table 1). No side effects of antigravity training were reported by any patient.

## 4. Discussion

We present the effect of a novel therapy to treat FoG in patients with PD. Our results show that a relatively low intensity training program provided benefit in several FoG scores and increased mobility manifested by lower TUG times with lower incidence of falls; patients also perceived benefit in other aspects of their gait such as balance/equilibrium, gait speed and larger steps. The high TUG observed at baseline and following the anti-gravity treadmill trainings are explained as we selected the most impaired patients from a cohort. Although it is possible that a patient with atypical parkinsonism was included, we carefully excluded those patients at baseline. It is unclear why the total MDS-UPDRS-III score improved at the end of therapy, although it is known that various motor symptoms may improve following treatment with physical therapy. The relatively low MoCA scores in our patients suggest that the machine can provide benefit even in cognitively impaired patients; whether patients with higher MoCA scores would obtain higher benefit from the therapy should be further investigated.

The pathophysiology of FoG is complex and not fully understood where a network of cortical and subcortical structures seems to dysfunction leading to a multilevel neuronal integration failure [14]. We hypothesize that attenuation of ground reaction forces provided by the machine may lead to better integration of neuronal motor circuits with the patient regaining certain motor abilities. It is also possible that patients may enhance neuronal compensatory mechanisms with improvement in gait [14]. The antigravity treadmill training has shown several benefits in children with cerebral palsy, including: improved muscle stiffness, decreased reflex hyperexcitability and enhanced corticostriatal tract function [15]. Moreover, improvement in dynamic balance and postural stability has also been observed in these patients [16]. These changes have been related to improved neuroplasticity [17,18]. Neuroplasticity in dopaminergic signaling with increased D2 receptor binding potential was suggested in one study of patients with early-stage PD who underwent intensive (non-antigravity) treadmill training [19]. Increased functional connectivity of diverse motor nodes has also been observed in patients undergoing lower extremity forced exercise resembling the effect of anti-parkinsonian medications [20,21].

Our study has limitations; we did not include a comparison group, for example patients undergoing treadmill training without the antigravity effect; this would help to clarify whether a placebo effect is involved in the improvement. A strength of this study is that we showed benefit in the most impaired patients, at the cost of reducing the external validity for less motor impaired patients. The current study is a pilot one that will help to plan future calculations in the number of patients included in a randomized trial. Considering the improvement in the mean TUG observed in this study (27%), a trial with 154 subjects would be necessary, considering a statistical power of 80%, and an alpha error of 0.05. Further studies should also help to clarify which is the ideal weight reduction or if this variable should be adapted to individual cases depending on the severity of the gait disorder or FoG. Video recordings before and after treatment are desirable; unfortunately, they only capture a moment in time and patients usually perform well while videotaped, decreasing the usefulness of this method; home-based assessments with wearable technology could be used in the future for objective evaluations of gait parameters in patients undergoing this therapy. Moreover, the reliability of the FoGQ and new-FoGQ has been questioned owing to the relatively high inter-rater reliability [22]; making necessary to include more reliable measures for FoG in further studies. Finally, although AlterG machines are relatively expensive, they can provide benefit for several patients with PD in a center devoted for physical therapy.

## 5. Conclusions

We present the effect of a novel rehabilitation therapy with an antigravity treadmill showing improvement in FoG and mobility in patients with PD. Further studies should clarify how the antigravity effect improves gait in PD and better define the training variables that can provide the maximal clinical benefit, as this therapy is quite easy and practical to apply for clinicians and rehabilitation technicians with acceptable compliance by patients with low risk of side effects.

## Figures and Tables

**Table 1:** Summary of clinical features of included patients.

	Mean ± S.D.
Age at evaluation (years)	72.68 ± 10.12
Evolution time of PD (years)	6.8 ± 5.4
Hoehn–Yahr stage	3.58 ± 0.69
MoCA score	23.3 ± 4.2
FoG relationship with levodopa	n (%)
Unknown/Unclear	5 (26.3)
Partially sensible	12 (63.2)
Resistant	2 (10.5)
Induced	0 (0)
CGIS-severity (at baseline)	n (%)
Moderate	14 (73.7)
Marked	4 (21.1)
Severe	1 (5.3)
CGIS-improvement (after treatment)	n (%)
Much better	3 (15.8)
Moderately better	13 (68.4)
Mildly better	2 (10.5)
No change	1 (5.3)
Aspects with perceived improvement	n (%)
Balance/equilibrium	17 (89.5)
Gait speed	16 (84.2)
Step amplitude	16 (84.2)
FoG	16 (84.2)

CGIS: Clinical Global Impressions Scale; FoG: freezing of gait; MoCA: Montreal Cognitive Assessment; PD: Parkinson´s disease.

**Table 2:** Baseline and post-therapy features of patients included in the study.

	Before Therapy	After Therapy	*p*-Value
**FOGQ**			
1.1. Gait at worst state	2.79 ± 0.63	2.21 ± 0.79	0.002
1.2. Gait difficulties affecting DA and independence	2.16 ± 0.96	2.05 ± 0.78	0.578
1.3. Frequency of FoG episodes	2.79 ± 1.13	2.16 ± 1.5	0.042
1.4. Duration of longest FoG episodes	1.84 ± 0.90	1.11 ± 1.20	0.005
1.5. Duration of FoG when initiating the first step	2.47 ± 0.77	1.95 ± 1.13	0.008
1.6. Duration of freezing when turning	1.95 ± 0.97	1.37 ± 1.06	0.053
Total score	14.05 ± 3.37	10.79 ± 4.70	0.001
**MDS-UPDRS Part II**			
Item 2.13 (FoG)	2.58 ± 1.12	1.58 ± 1.12	<0.001
**MDS-UPDRS Part III**			
Item 3.10 (gait)	2.16 ± 0.90	1.63 ± 0.89	0.004
Item 3.11 (FoG)	0.79 ± 1.03	0.32 ± 0.75	0.016
Total score	31.79 ± 12.61	28.16 ± 13.38	0.004
**TUG test**			
1st attempt	27.06 ± 17.39	21.12 ± 11.13	0.027
2nd attempt	26.55 ± 15.97	18.98 ± 10.19	0.003
3rd attempt	25.49 ± 15.27	18.95 ± 10.34	0.007
Mean	26.01 ± 15.57	18.96 ± 10.20	0.004
**Number of falls**	3.26 ± 10.27	1.37 ± 3.77	0.243

DA: daily activities; FoGQ-B: freezing of gait questionnaire part-B; MDS-UPDRS: Movement Disorders Society Unified Parkinson’s Disease Rating Scale. TUG: Timed Up & Go test; Higher score means worse clinical state in all these tests.
